# Supplementary material for: Resolving the full spectrum of human genome variation using Linked-Reads
Source: Genome Res. 2019 Apr;29(4):635–45. doi: 10.1101/gr.234443.118 (PMC6442396; doi:10.1101/gr.234443.118)
Supplement: Supplemental Material [file supp_gr.234443.118_Supplemental_longranger-2.2.zip › longranger-2.2.tar.bz2!/lr2.2-public/lib/stan/cmdstan/stan/doc/api/html/newton_8hpp_source.html]

Stan: src/stan/optimization/newton.hpp Source File


|  |  |
| --- | --- |
|  | Stan  2.5.0  probability, sampling & optimization |


- Main Page
- Related Pages
- Namespaces
- Classes
- Files

- File List
- File Members

All Classes Namespaces Files Functions Variables Typedefs Enumerations Enumerator Friends Macros Pages

- src
- stan
- optimization

newton.hpp

Go to the documentation of this file.

1 #ifndef STAN\_\_OPTIMIZATION\_\_NEWTON\_HPP

2 #define STAN\_\_OPTIMIZATION\_\_NEWTON\_HPP

3

4 #include <Eigen/Dense>

5 #include <Eigen/Cholesky>

6 #include <Eigen/Eigenvalues>

7

8 #include <stan/model/util.hpp>

9

10 namespace stan {

11

12  namespace optimization {

13

14  typedef Eigen::Matrix<double, Eigen::Dynamic, Eigen::Dynamic> matrix\_d;

15  typedef Eigen::Matrix<double, Eigen::Dynamic, 1> vector\_d;

16

17  namespace {

18  // Negates any positive eigenvalues in H so that H is negative

19  // definite, and then solves Hu = g and stores the result into

20  // g. Avoids problems due to non-log-concave distributions.

21  void make\_negative\_definite\_and\_solve(matrix\_d& H, vector\_d& g) {

22  Eigen::SelfAdjointEigenSolver<matrix\_d> solver(H);

23  matrix\_d eigenvectors = solver.eigenvectors();

24  vector\_d eigenvalues = solver.eigenvalues();

25  vector\_d eigenprojections = eigenvectors.transpose() \* g;

26  for (int i = 0; i < g.size(); i++) {

27  eigenprojections[i] = -eigenprojections[i] / fabs(eigenvalues[i]);

28  }

29  g = eigenvectors \* eigenprojections;

30  }

31  }

32

33  template <typename M>

34  double newton\_step(M& model,

35  std::vector<double>& params\_r,

36  std::vector<int>& params\_i,

37  std::ostream\* output\_stream = 0) {

38  std::vector<double> gradient;

39  std::vector<double> hessian;

40

41  double f0

42  = stan::model::grad\_hess\_log\_prob<true,false>(model,

43  params\_r, params\_i,

44  gradient, hessian);

45  matrix\_d H(params\_r.size(), params\_r.size());

46  for (size\_t i = 0; i < hessian.size(); i++) {

47  H(i) = hessian[i];

48  }

49  vector\_d g(params\_r.size());

50  for (size\_t i = 0; i < gradient.size(); i++)

51  g(i) = gradient[i];

52  make\_negative\_definite\_and\_solve(H, g);

53 // H.ldlt().solveInPlace(g);

54

55  std::vector<double> new\_params\_r(params\_r.size());

56  double step\_size = 2;

57  double min\_step\_size = 1e-50;

58  double f1 = -1e100;

59

60  while (f1 < f0) {

61  step\_size \*= 0.5;

62  if (step\_size < min\_step\_size)

63  return f0;

64

65  for (size\_t i = 0; i < params\_r.size(); i++)

66  new\_params\_r[i] = params\_r[i] - step\_size \* g[i];

67  try {

68  f1 = stan::model::log\_prob\_grad<true,false>(model,

69  new\_params\_r,

70  params\_i, gradient);

71  } catch (std::exception& e) {

72  // FIXME: this is not a good way to handle a general exception

73  f1 = -1e100;

74  }

75  }

76  for (size\_t i = 0; i < params\_r.size(); i++)

77  params\_r[i] = new\_params\_r[i];

78

79  return f1;

80  }

81

82  }

83

84 }

85

86 #endif

stan::agrad::gradient

void gradient(const F &f, const Eigen::Matrix< double, Eigen::Dynamic, 1 > &x, double &fx, Eigen::Matrix< double, Eigen::Dynamic, 1 > &grad\_fx)

Calculate the value and the gradient of the specified function at the specified argument.

**Definition:** autodiff.hpp:93

stan::agrad::fabs

fvar< T > fabs(const fvar< T > &x)

**Definition:** fabs.hpp:18

stan::optimization::newton\_step

double newton\_step(M &model, std::vector< double > &params\_r, std::vector< int > &params\_i, std::ostream \*output\_stream=0)

**Definition:** newton.hpp:34

stan::optimization::matrix\_d

Eigen::Matrix< double, Eigen::Dynamic, Eigen::Dynamic > matrix\_d

**Definition:** newton.hpp:14

util.hpp

stan::optimization::vector\_d

Eigen::Matrix< double, Eigen::Dynamic, 1 > vector\_d

**Definition:** newton.hpp:15

stan::math::e

double e()

Return the base of the natural logarithm.

**Definition:** constants.hpp:86

stan::agrad::hessian

void hessian(const F &f, const Eigen::Matrix< double, Eigen::Dynamic, 1 > &x, double &fx, Eigen::Matrix< double, Eigen::Dynamic, 1 > &grad, Eigen::Matrix< double, Eigen::Dynamic, Eigen::Dynamic > &H)

**Definition:** autodiff.hpp:194


---

[ Stan Home Page ]

*© 2011–2014,
Stan Development Team.*
